# Influence of Active Recovery on Cardiovascular Function During Ice Hockey

**DOI:** 10.1186/s40798-015-0026-8

**Published:** 2015-08-28

**Authors:** Jamie F. Burr, Joshua T. Slysz, Matthew S. Boulter, Darren E. R. Warburton

**Affiliations:** 1 Human Performance Laboratory, University of Guelph, 50 Stone Road East, Guelph, Ontario N1G 2W1 Canada; 2 Human Performance and Health Research Laboratory, University of PEI, Charlottetown, Canada; 3 Cardiovascular Physiology and Rehabilitation Laboratory, University of British Columbia, Vancouver, Canada

**Keywords:** Impedance cardiography, Performance, Cardiac risk, Venous return, Sprint

## Abstract

**Background:**

Ice hockey is a popular sport comprised of high-intensity repeated bouts of activity. Light activity, as opposed to passive rest, has been shown to improve power output in repeated sprinting and could potentially help to offset venous pooling, poor perfusion, and the risk of an ischemic event. The objective of our study was, thus, to examine the efficacy of low-intensity lower body activity following a simulated hockey shift for altering hemodynamic function.

**Methods:**

In a cross-over design, 15 healthy hockey players (23 ± 1 years, 54 ± 3 mL/kg/min) performed two simulated hockey shifts. In both conditions, players skated up to 85 % of age-predicted heart rate maximum, followed by either passive recovery or active recovery while hemodynamic measures were tracked for up to 180 s of rest.

**Results:**

Light active recovery within the confines of an ice hockey bench, while wearing skates and protective gear, was effective for augmenting cardiac output (an average of 2.5 ± 0.2 L/min, *p* = 0.03) at 45, 50, and 120 s. These alterations were driven by a sustained elevation in heart rate (12 bpm, *p* = 0.05) combined with a physiological relevant but non-significant (11.6 mL, *p* = 0.06) increase in stroke volume.

**Conclusions:**

Standing and pacing between shifts offers a realistic in-game solution to help slow the precipitous drop in cardiac output (heart rate and stroke volume) that typically occurs with passive rest. Prolonging the duration of an elevated cardiac output further into recovery may be beneficial for promoting recovery of the working skeletal muscles and also avoiding venous pooling and reduced myocardial perfusion.

**Key Points:**

- Evidence that light activity in the form of standing/pacing is effective for maintaining cardiac output, and thus venous return
- Increased cardiac output and venous return may help reduce the chances of poor perfusion (ischemia) and could also promote recovery for performance
- This is a simple, low-risk, intervention demonstrated for the first time to work within the confines of a player’s bench while wearing hockey gear

## Background

The sport of ice hockey is characterized by stop and start play, with repeated high-intensity exercise interspersed with seated rest on the bench. At the highest level, on-ice shifts typically last between 30 and 60 s [1], with a work to rest ratio of 1:3. This differs somewhat from recreational “old-timer” hockey wherein the work to rest ratio is closer to 1 owing to longer less intense shifts and fewer players per team [2–4].

During competitive hockey, on-ice heart rates (HR) are often sustained as high as 85 % max and have been reported to peak >90 % [4]. Atwal et al. (2002) employed Holter monitoring to demonstrate HR ranges between 85 and 100 % of age-predicted max during recreational hockey [5], and our own lab has (unpublished) data confirming these results in recreational male hockey players (65 ± 6 years), with the average shift representing 97 ± 8 % of age predicted HRmax, and peaks of 103 ± 7 % (reflecting the variability around estimations of max HR). Goodman et al. have recently demonstrated peak on-ice HRs that actually exceeded the objectively measured maximal values observed during laboratory fitness tests (i.e., 107 % of laboratory max) [2]. Given these physical demands of ice hockey, it is logical that the level of competitive play and “success” in hockey is associated with well-developed physiological attributes [6–8]. Potentially compounding the high intensity of play is the fact that many older recreational hockey players also have poorly controlled cardiovascular risk factors [5, 9], and thus, it is perhaps not surprising that hockey is often suggested as a high-risk sport for this population. There is compelling evidence that the risk for adverse exercise-related event (such as sudden cardiac death) increases significantly in vigorous activities greater than 6 metabolic equivalents (METs) (with hockey reported to be ≥8 METs [10]), and this risk is markedly increased in previously inactive or untrained individuals [11, 12].

The intermittent nature of hockey, which requires participants to perform particularly stressful exercise followed by relative inactivity between shifts, represents an interesting cardiovascular challenge for both the competitive player and recreational “old-timer” alike. Given the evidence that higher exercise intensities lead to greater metabolite-induced vasodilation post-exercise, this is particularly salient in the sport of ice hockey [13]. For the competitive athlete, the challenge of maintaining optimal performance is paramount, whereas the avoidance of cardiac ischemia may be a primary goal for the prevention of adverse cardiac events in the aged athlete.

Given that the circulatory system can be modeled as a simple closed-circuit loop with a single pump, venous return must equal cardiac output (*Q*). It also follows that augmented venous return would thus ensure that *Q* is optimized, thus potentially augmenting both central and peripheral blood supply for either performance or health. It is for this reason that exercisers are commonly encouraged to include a “cool-down” period of light exercise following physical activity in an attempt to slow the decline in HR and stroke volume (SV), by maintaining a light cardiovascular demand and decreasing venous pooling. However, in ice hockey, it is often not possible for athletes to slowly reduce the exercise intensity through low-intensity skating following each shift, as the game play continues and the athlete is within the confines of the player’s bench. Nevertheless, participants are recommended to find alternative methods of promoting recovery and venous return following the cessation of vigorous exercise to ensure adequate cerebral, musculoskeletal, and cardiac flow.

Although standing or walking in place between shifts in hockey has been suggested for those who may be at an increased risk of cardiovascular event [14], empirical evidence demonstrating the efficacy of such actions on cardiac responses for hockey players is lacking. Investigations of active versus passive recovery using cycling exercise have demonstrated active recovery to maintain *Q* by maintaining a higher SV and HR compared to passive recovery [15, 16] likely through alterations in systemic vascular resistance to maintain blood and perfusion pressure [17]. However, the efficacy of using the muscle pump during ice hockey could be greatly compromised considering the restrictions of the player’s bench and the athlete’s ability to contract the musculature of their legs. In particular, lower leg contractions involving dorsi and plantar flexion may be limited by the stiff upper boot of a hockey skate. It is also possible that related hockey-specific environmental factors (i.e., equipment, environment) might affect the ecological validity of implementing this technique for altering cardiac and hemodynamic response outside of a laboratory setting.

The objective of our study was, thus, to examine the efficacy of changing body posture (stand vs. sit) and including low-intensity lower body activity following a simulated hockey shift for altering hemodynamic function. We hypothesized that standing and pacing between shifts would assist in the maintenance of *Q* by augmenting venous return and SV by way of peripheral muscle pump action and through a sustained increase in HR.

## Methods

We recruited 15 young (22.5 ± 0.9 years) male hockey players from the varsity men’s hockey team to participate in a cross-over controlled study. Participants were of average build and fitness for competitive hockey players [7] as can be seen from the descriptive participant characteristics presented in Table 1. Player fitness was measured during a pre-season physical fitness combine, with VO_2_ estimated from maximal performance on a widely used and validated field-based 20-m shuttle run, for which methods have been described in detail elsewhere [18]. Prior to data collection, all participants provided written informed consent, and this study was approved by the university’s research ethics board (ref #6005349) for investigations involving human participants, in accordance with the Helsinki Declaration of 1975, as revised in 2008.

**Table 1:** Descriptive participant statistics

	$\overline{X}$	SD
Age (year)	22.5	0.9
Height (cm)	181.8	6.9
Weight (kg)	89.0	6.1
BMI (kg/m^2^)	26.9	1.2
Est. VO_2_max (mL/kg/min)	53.7	3
Systolic BP (mmHg)	119	2
Diastolic BP (mmHg)	77	3
Est. HRmax (bpm; 220 − age)	198	0.9
Body surface area (m^2^)	2.1	0.1

### Cardiovascular Measurements

Cardiovascular hemodynamic measures were recorded for each player using impedance cardiography (Physioflow Enduro, Manatec Biomedical, France) with a telemetric signal relayed wirelessly to a dedicated computer, housed rink-side in the player’s bench. Thoracic bio-impedance is a non-invasive and portable method of determining *Q* and has been shown to have good agreement with thermodilution techniques [19] at rest and also with the direct Fick method during incremental exercise to maximum exertion [20]. The Physioflow impedance cardiography (ICG) device that we employed differs from conventional ICG in that there is less reliance on the baseline thoracic impedance signal (resistance ZO) for transformation during exercise; instead, a pulsatile waveform representing the sensed impedance signal (delta Z) is processed and analyzed with its derivative over time. This signal morphology filter improves the signal to noise ratio and reduces artifact introduced by exercise, making it ideally suited for our purposes. During testing, participants wore full hockey gear, with the exception of shoulder pads so as not to interfere with the electrodes placed on the upper chest and back. Prior to testing, the participant’s skin was cleaned, shaved, and abraded (Nu-prep), and surface electrodes (SkinTact FS-50) were placed according to the landmarks recommended by the manufacturer for exercise testing. On the hockey bench adjacent to the ice surface, the participant was instructed to sit quietly for 5–10 min prior to the measurement of blood pressure with a standard sphygmomanometer for calibration of the ICG device. Blood pressure was re-evaluated and corrected for ICG measures periodically during recovery.

### Experimental Protocol

Baseline hemodynamic data was collected at rest on each participant prior to undertaking two skate-rest conditions. In condition 1, participants skated shuttles back and forth on the ice for 60 s, bringing their heart rate up to the typical in-game intensity [3, 4] of approximately 85 % of maximum, followed by a seated 180-s rest on the bench. In condition 2, participants again skated for 60 s at the same intensity followed by a 180-s “active” recovery on the bench. The active recovery consisted of standing and pacing on the spot or pacing within the confines of the bench that would normally be afforded to a player during a real game situation. Every participant performed both conditions consecutively in a randomized order. As such, both posture and muscular pump action were altered and compared in a cross-over design. Following baseline calibration, a short warm-up was permitted, after which time all exercise and rest was standardized to the above-noted work to rest ratio. ICG data was collected and analyzed using 15-s averages with measurements considered at 15, 30, 45, 60, 120, and 180 s post exercise.

#### Statistical Analysis

We employed a two-way repeated measures ANOVA comprising a 2 (recovery condition; passive or active) × 6 (time point) model. Time effects were examined using Bonferroni adjusted post hoc pairwise comparisons, and repeated measures *t* tests were used to compare mean differences between intervention groups at select time points, when indicated by main effects or interaction. Normality was confirmed using a Shapiro-Wilk test, and Greenhouse-Geisser correction was applied for interpretation of ANOVAs when sphericity was violated according to Mauchly’s test. Exertion at peak exercise (% predicted HRmax) and hemodynamic responses were compared between conditions using paired sample *t* tests. All stats were performed using SPSS v21.0 and an alpha of *p* < 0.05 was selected a priori. All results are presented as mean ± SD unless otherwise noted.

## Results

**Fig. 1:**
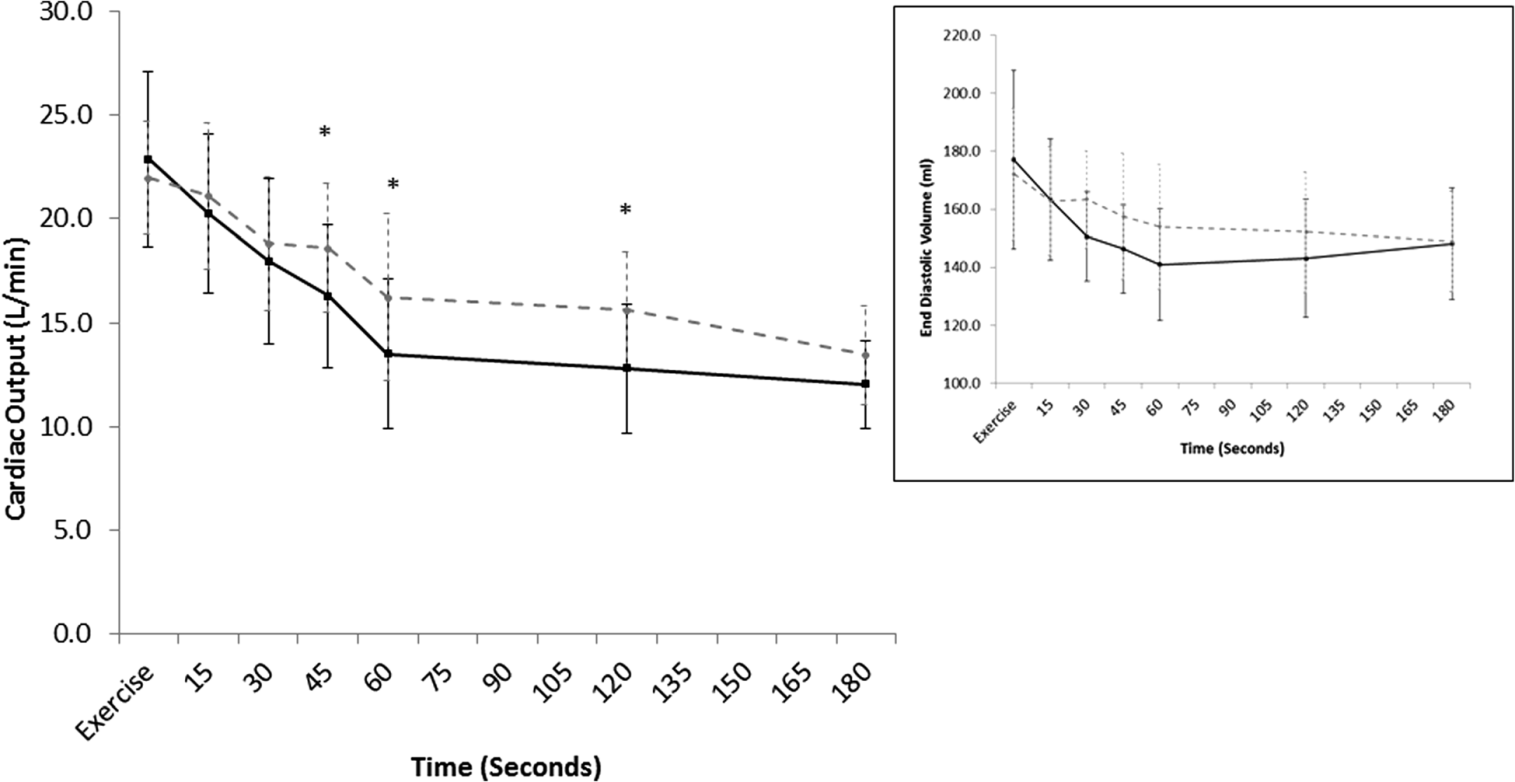
A comparison of the cardiac output measured by impedance cardiography during skating exercise and following 15–180 s of recovery in hockey players who passively sat on the bench (*solid line*) or performed light activity (*dashed line*) by standing and rocking on feet. (*Inset*) End-diastolic volumes (EDV) during the same period. Statistical significance was not met for EDV which had larger variance about the mean, but the similarity in the shape of these curves is notable. * *p*<0.05

**Fig. 2:**
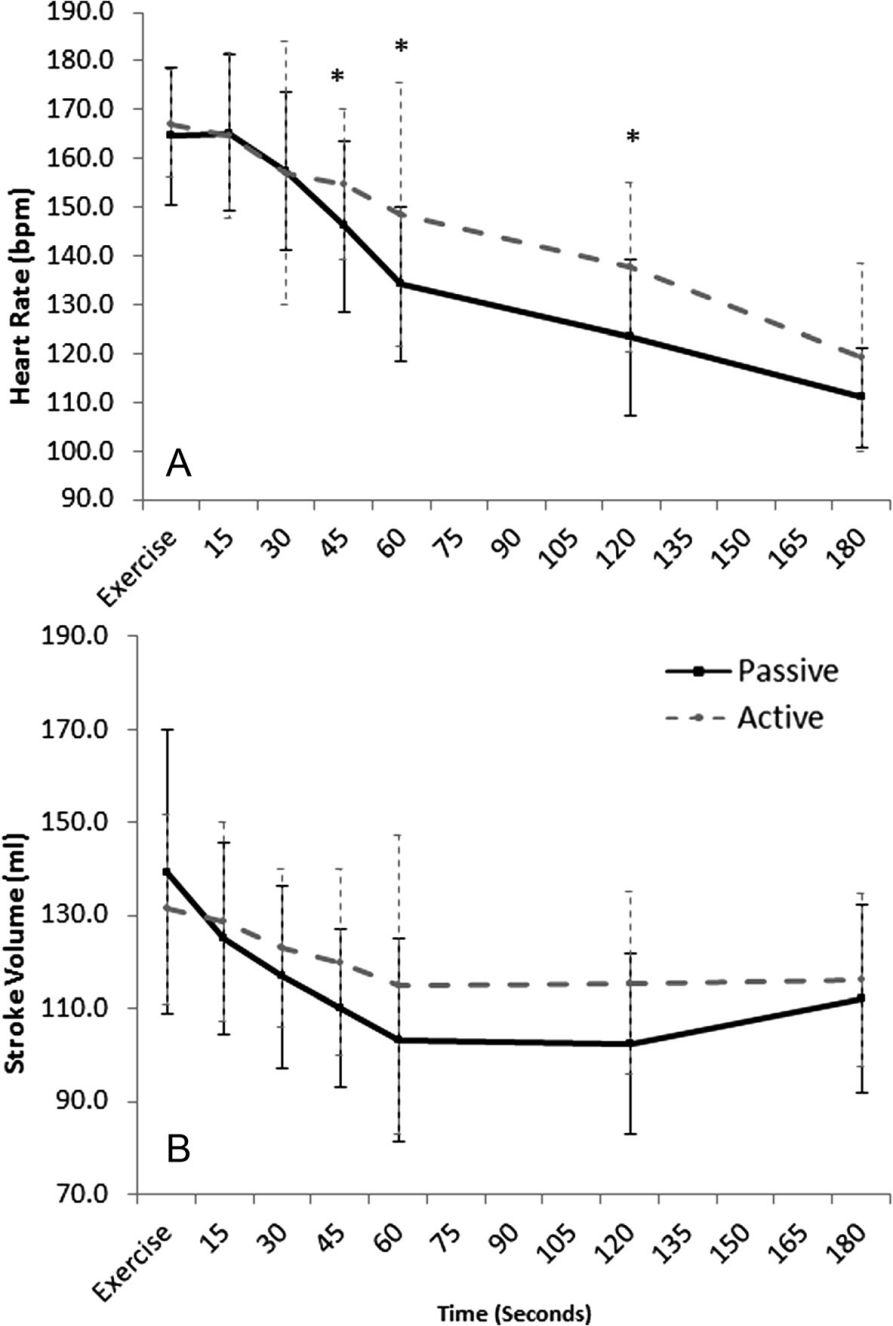
Independent graphical representation of each of the components of cardiac output. These figures demonstrate the recovery response of heart rate (**a**) and stroke volume (**b**) following skating exercise in hockey players who either sat passively (*solid line*) or stood and paced (*dashed line*) at the bench. * *p*<0.05

There was no difference in the level of exertion reached during a simulated shift between recovery conditions, with players skating up to a mean intensity of 83 ± 7 % (197 ± 1 bpm) and 84 ± 6 % (198 ± 1 bpm) of age-predicted HRmax for the passive and active trials, respectively. Similarly, no differences existed in hemodynamic variables at the end of the exercise period immediately prior to the initiation of recovery, which for the purposes of this study represent baseline values as the recovery phase was the time period of interest. As can be seen in Fig. 1, an interaction between time and recovery condition was observed for *Q* (*p* = 0.03), such that the difference in *Q* between groups increased throughout rest, reaching a statistically different value of approximately 2.5 L/min at 45 s, which was maintained at 60 and 120 s post exercise before values began to converge by the final measurement at 180 s. Examination of the contributing factors to *Q* (Fig. 2) revealed alterations over time for both HR (*p* < 0.001) and SV (*p* = 0.004) as they returned toward resting levels. There was also a main effect on HR by condition (*p* = 0.05) with differences between groups presenting at 45 s of recovery and persisting until through the 120-s measurement. Although a clear trend was apparent for alterations in SV by recovery condition, this did not reach significance (*p* = 0.06). Importantly, the changes in SV would be considered to be of physiological importance [21]. Related hemodynamic variables are presented in Table 2, where it can be seen that only cardiac index (expressed relative to body size) and left heart work index differed between recovery conditions.

**Table 2:** Hemodynamic variables associated with cardiovascular function and recovery following a high-intensity short-duration simulated hockey shift

	Time (s)	Cardiac index (L/min/m^2^)	Ejection fraction (%)	End-diastolic volume (mL)	Contractility index (no unit)	Left cardiac work index (kg.m/m^2^)
Passive	Exercise	10.8 ± 1.8	78.6 ± 7.1	177 ± 30.8	372 ± 178.9	12.8 ± 2.3
	15	9.5 ± 1.7	76.2 ± 9	163.3 ± 20.9	314.9 ± 129	11.5 ± 2.2
	30	8.4 ± 1.8	74.7 ± 11.4	150.5 ± 15.4	273 ± 97.5	10 ± 2.3
	45	7.6 ± 1.6	75 ± 9.1	146.3 ± 15.2	264.1 ± 74.2	9 ± 2.1
	60	6.3 ± 1.6	70.6 ± 12.5	140.9 ± 19.2	220.6 ± 94.1	7.5 ± 2.1
	120	6 ± 1.4	71.6 ± 11.2	143 ± 20.2	229.2 ± 84.9	7.1 ± 1.7
	180	5.6 ± 0.9	72.6 ± 11.6	148.2 ± 19.2	249.1 ± 89.6	6.9 ± 0.9
Active	Exercise	10.3 ± 1.1	76.7 ± 8.5	172.1 ± 22.6	324.2 ± 147	12.3 ± 1.5
	15	9.9 ± 1.5	78.3 ± 9	162.8 ± 18.6	350.9 ± 154.7	11.5 ± 1.7
	30	8.8 ± 1.3	75.2 ± 10.6	163.4 ± 16.7	290.2 ± 116.9	10.5 ± 1.8*
	45	8.7 ± 1.3*	76.5 ± 9.6	157.5 ± 21.7	301 ± 120	10.1 ± 1.4*
	60	10.3 ± 8.5*	81.1 ± 14.9	154 ± 21.5	293.8 ± 111.3	9.8 ± 1.5*
	120	7.3 ± 1.1*	74.4 ± 10.2	152.1 ± 20.8	261.6 ± 79.5	8.9 ± 1.1
	180	6.3 ± 1	76.2 ± 7.7	148.8 ± 17.3	296.4 ± 115.2	7.7 ± 1.2
	Condition	*p* = 0.01	*p* = 0.09	*p* = 0.266	*p* = 0.09	*p* = 0.003
	Time	*p* < 0.00	*p* = 0.485	*p* = 0.14	*p* = 0.07	*p* < 0.00
	Interaction	*p* = 0.03	*p* = 0.222	*p* = 0.464	*p* = 0.235	*p* = 0.02

*Significantly different *p* < 0.05 from passive condition for select time point

*p* values across the bottom represent main effects and interactions across groups and time

## Discussion

Altering body posture from sitting to standing and engaging the muscle mass of the lower legs as a recovery strategy following a simulated hockey shift was found to be effective for maintaining *Q* and slowing the precipitous drop in *Q* that occurs with the abrupt cessation of exercise. This paper presents novel evidence that standing and pacing on the bench between shifts may represent an easily adopted and viable alternative to light skating to “cool down” slowly between shifts, when a player must leave the ice. This may have important implications for the high-performance athlete and aging recreational hockey player alike, both of whom face potential challenges that stem from insufficient *Q* between shifts while the body attempts to recover from a previous bout of stressful activity.

The current data supported our hypothesis regarding the overall effects on *Q*; however, the hypothesized mechanisms of this elevation were not fully supported, as elevations in SV did not reach significance. As can be observed from the graphical representation of data in Fig. 2, the elevated response of SV and HR in the active recovery condition was temporally similar; however, the larger variance around the measure of SV compared to HR almost certainly played a role in reducing the chances of reaching statistical significance (*p* = 0.06). In the current investigation, blood pressure was used to calibrate the ICG unit; however, a continuous BP signal was not recorded throughout exercise or recovery owing to the “field-based” nature of data collection, which is a limitation worth noting. Alterations in HR for time points 45–120 s, which significantly differed between active and passive recovery conditions, had a mean difference of 12 ± 3 bpm. At the matched time points, the mean alteration in SV was 12 ± 6 mL/beat, thus suggesting that the change in SV actually represented a slightly greater relative deviation from baseline at 8.8 % compared to HR at 7.5 %. Notably, alterations did not occur within the first 30 s of recovery, which likely reflects the time necessary for the cardiovascular system to adjust to the changing exercise demands. By 180 s, difference between the active and passive conditions started to disappear, and this likely relates to the fact that the young, fit hockey players used in the current investigation were conditioned to recover in approximately 3–4 min of rest. This effect may be amplified (at both the beginning and end of the rest period) in less fit athletes, or by altering the intensity of the active recovery condition, which was intentionally light in the current investigation.

### Cardiovascular Implications for Performance

There is convincing evidence from cycle ergometry models demonstrating significant decrements in repeated sprint ability when athletes recover passively between work bouts, as opposed to engaging in a light “active” recovery [22, 23]. It is suggested that this effect is primarily driven by ATP repletion and pH recovery, both of which are affected by *Q*/blood flow [22] through alterations in O_2_ delivery and the maintenance of metabolite (H^+^) gradients between the passing blood and local muscle tissue [24, 25]. Corresponding to the temporal demands of a hockey shift, evidence clearly demonstrates that sprints of 15–30-s duration, separated by rests of 3–4 min, show benefits to mechanical power output when light activity is substituted for passive rest, particularly in the first 10–15 s [22, 23]. As such, it would appear that competitive athletes may benefit from improved performance as a result of better recovery, but direct tests of skating speed, power, and longer term in-game adoption of this strategy are warranted.

### Cardiovascular Implications for Health

By contrast, blood flow may have different, yet equally important, implications for the older recreational hockey player given the effects on central, rather than peripheral, circulation. Despite the well-accepted understanding that the risk reward of exercise greatly favors participation [11], it is undeniable that exercise can act as a trigger for a myocardial infarction or sudden cardiac death in the susceptible myocardium [12, 26]. Among the factors that might act to trigger these events in-game, or immediately after exercise, are the decrease in *Q* and relatively slow compensatory vasoconstriction of leg vasculature [27, 28]. This in turn can affect venous return, *Q*, and coronary perfusion, particularly in the vulnerable heart wherein supply and demand mismatches may be exacerbated. Prolonged intense exercise in particular (as often occurs in many recreational hockey games wherein there are suboptimal numbers of players per team to offer regular shift changes) can worsen these effects. If this type of prolonged vigorous play also leads to significant heat stress and dehydration [29], the associated reductions in blood plasma, coagulatory factors, and thermoregulatory fluid shifts have the potential to further compound the supply and demand imbalance through a reduction in stroke volume and compensatory increase in heart rate [30]. Prolonging an elevated *Q* further into recovery may be beneficial for promoting recovery of the working skeletal muscles and also avoiding venous pooling and reduced myocardial perfusion, particularly in persons with compromised coronary artery flow.

#### Mechanistic Implications

For the high-performance athlete, the mechanism for maintaining an increased *Q* may be of little consequence, as perfusion of the working muscles is augmented nonetheless, and improvements in blood flow-driven blood/tissue gradients (for the transfer of O_2_, nutrients, and waste products) are established regardless of the underlying hemodynamic alterations. However, for the aging hockey player with a potentially susceptible myocardium, the observed mechanisms supporting an elevated *Q* could be further optimized for avoiding a supply and demand imbalance. This may be of greater importance for some individuals more than others given the clearly divergent responses in SV evident in the large variability in individual response, but it should be noted that the current population comprised exclusively of young ostensibly healthy men, who were of high fitness. The response of older potentially susceptible participants may be more homogenous in its response [31]. In practice, myocardial supply would be enhanced by reductions in HR, which would prolong diastolic filling time during which coronary arteries supply oxygenated blood to the cardiac tissue itself [32]. A current trend in sports performance apparel includes the wearing of compression garments to improve both performance and recovery through alterations in blood flow. If external compression devices are capable of promoting improved venous return [33], there may be a yet unexplored role for such garments in helping to bridge the gap between the benefits of activity for promoting long-term health and fitness and the acute risk posed during a given exercise session in a potentially at-risk population. External compression combined with light activity could also potentially alter the effective time-course of the observed recovery effects and should be further explored.

## Conclusions

Standing and pacing on the hockey bench between shifts offers a realistic in-game solution to help slow the rapid drop in cardiac output (heart rate and stroke volume) that typically occurs with passive rest on the bench between hockey shifts. As such, adoption of an on-bench practice of standing recovery offers an easily implemented and low-cost solution with low participant risk, but a potentially high benefit.

## Abbreviations

HR: heart rate
*Q*: cardiac output
ICG: impedance cardiography
SV: stroke volume
